# Phylogeography of the Sunda pangolin, *Manis javanica*: Implications for taxonomy, conservation management and wildlife forensics

**DOI:** 10.1002/ece3.10373

**Published:** 2023-08-15

**Authors:** Frankie T. Sitam, Milena Salgado‐Lynn, Azroie Denel, Elisa Panjang, Ross McEwing, Amanda Lightson, Rob Ogden, Nur Alwanie Maruji, Nurhartini Kamalia Yahya, Cosmas Ngau, Noor Azleen Mohd Kulaimi, Hartini Ithnin, Jeffrine Rovie‐Ryan, Mohd Soffian Abu Bakar, Kyle M. Ewart

**Affiliations:** ^1^ Department of Wildlife and National Parks (DWNP/PERHILITAN) National Wildlife Forensic Laboratory (NWFL) Kuala Lumpur Malaysia; ^2^ Danau Girang Field Centre (DGFC) Kota Kinabalu Malaysia; ^3^ Wildlife Health, Genetic and Forensic Laboratory (WHGFL) Kota Kinabalu Malaysia; ^4^ Organisms and Environment Division, Cardiff School of Biosciences Cardiff University Cardiff UK; ^5^ Sarawak Forestry Corporation (SFC) Kuching Malaysia; ^6^ TRACE Wildlife Forensics Network Edinburgh UK; ^7^ Royal (Dick) School of Veterinary Studies and the Roslin Institute University of Edinburgh Edinburgh UK; ^8^ Sabah Wildlife Department (SWD) Kota Kinabalu Malaysia; ^9^ Universiti Malaysia Sarawak (UNIMAS) Kuching Malaysia; ^10^ School of Life and Environmental Sciences University of Sydney Sydney New South Wales Australia

**Keywords:** conservation genetics, *Manis javanica*, phylogenetics, phylogeography, Sundaland, wildlife forensics

## Abstract

The Sunda pangolin (*Manis javanica*) is the most widely distributed Asian pangolin species, occurring across much of Southeast Asia and in southern China. It is classified as Critically Endangered and is one of the most trafficked mammals in the world, which not only negatively impacts wild Sunda pangolin populations but also poses a potential disease risk to other species, including humans and livestock. Here, we aimed to investigate the species' phylogeography across its distribution to improve our understanding of the species' evolutionary history, elucidate any taxonomic uncertainties and enhance the species' conservation genetic management and potential wildlife forensics applications. We sequenced mtDNA genomes from 23 wild Sunda pangolins of known provenance originating from Malaysia to fill sampling gaps in previous studies, particularly in Borneo. To conduct phylogenetic and population genetic analyses of Sunda pangolins across their range, we integrated these newly generated mitochondrial genomes with previously generated mtDNA and nuclear DNA data sets (RAD‐seq SNP data). We identified an evolutionarily distinct mtDNA lineage in north Borneo, estimated to be ~1.6 million years divergent from lineages in west/south Borneo and the mainland, comparable to the divergence time from the Palawan pangolin. There appeared to be mitonuclear discordance, with no apparent genetic structure across Borneo based on analysis of nuclear SNPs. These findings are consistent with the ‘out of Borneo hypothesis’, whereby Sunda pangolins diversified in Borneo before subsequently migrating throughout Sundaland, and/or a secondary contact scenario between mainland and Borneo. We have elucidated possible taxonomic issues in the Sunda/Palawan pangolin complex and highlight the critical need for additional georeferenced samples to accurately apportion its range‐wide genetic variation into appropriate taxonomic and conservation units. Additionally, these data have improved forensic identification testing involving these species and permit the implementation of geographic provenance testing in some scenarios.

## Graphical abstract

The Sunda pangolin (*Manis javanica*), classified as Critically Endangered, is one of the most trafficked mammals in the world. Here, we perform a phylogeographic assessment of the Sunda pangolin across its Sundaland distribution. We identify an evolutionarily distinct mtDNA lineage in north Borneo and mitonuclear discordance across parts of the species' range, which together have several conservation genetic, wildlife forensic and taxonomic implications.

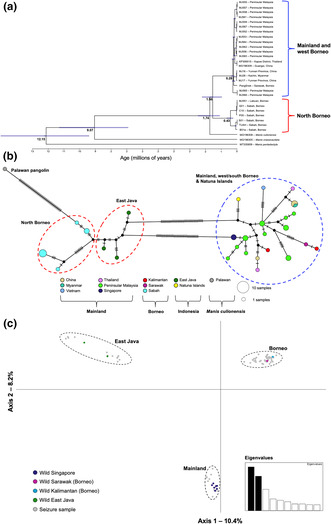

## 1 INTRODUCTION

Pangolins are a group of scaly mammals belonging to Manidae, the only extant family of Pholidota. All eight species of extant pangolin are highly sought after for consumption of their meat and for traditional medicinal use of their scales, and are considered the most highly trafficked mammal (Thomson & Fletcher, 2020). Between 2014 and 2018, pangolins accounted for a staggering 13.9% of the monetary value among all seizures of protected wildlife, including plants (UNODC, 2020). All eight species of pangolin were uplifted to CITES Appendix I in 2016, prohibiting any international trade of the group.

The Sunda pangolin (*Manis javanica* Desmarest, 1822) has the widest distribution among the four Asian pangolin species, occurring across much of Southeast Asia. Sunda pangolins are classified as Critically Endangered (IUCN Red List of Threatened Species; Challender et al., 2019), primarily a consequence of high levels of exploitation, with hundreds of thousands of individuals being killed for local consumption or the wildlife trade over the past few decades. The proliferation of trade in Sunda pangolins also poses a possible disease risk to humans, livestock and other wildlife populations. The species is known to harbour a variety of infectious agents (Barton et al., 2022; Lam et al., 2020; Liu et al., 2019; Nga et al., 2022; Peng et al., 2021; Shi et al., 2022; Wicker et al., 2020; Xiao et al., 2020), at least one of which has the capability to infect other species (Guo et al., 2022). Several viruses have been detected in multiple seized Sunda pangolins (Lam et al., 2020; Shi et al., 2022; Xiao et al., 2020), demonstrating a potential disease transmission pathway along the wildlife trade network, although there is no substantive evidence for the proposition that pangolins were an intermediate host in the spread of SARS‐Cov‐2 (Banerjee et al., 2021).

Despite its risk of imminent extinction, prevalence in cases of wildlife trafficking, and disease transmission risk, there remain significant gaps in our knowledge of the species' biology, including its evolutionary history and phylogeography. The Sunda pangolin is distributed from southwest China to Singapore, and inhabits several Southeast Asian islands including Borneo, Sumatra and Java (Chong et al., 2020). Sundaland (i.e. the region encompassing Borneo, Sumatra, Java and the Malay Peninsular) has experienced a complex geological and climatic history, particularly during the Plio‐Pleistocene (Voris, 2000), which has likely played an important role in shaping the phylogeography of Sunda pangolin. Nash et al. (2018) characterized three genetic lineages of Sunda pangolin putatively corresponding to Borneo, Java and Singapore/Sumatra populations, and found various levels of introgression between lineages. Subsequently, Hu, Hao, et al. (2020) delineated two Sunda pangolin lineages that diverged approximately 300 thousand years ago: one comprising individuals from the mainland, and one comprising individuals from Southeast Asian islands (with some mainland individuals). However, sampling gaps in these previous studies, particularly in Borneo (only four reference specimens from Borneo have been sequenced; Mason et al., 2019; Nash et al., 2018), obscures the species' phylogeography. In addition, numerous divergent haplotype clusters of unknown origin have been detected in various pangolin seizures (Gao et al., 2020; Zhang et al., 2015), one of which has even been suggested to represent a new species (Hu, Roos, et al., 2020). Evidently, more work is required to characterize the distribution of genetic diversity throughout the Sunda pangolin range.

An improved understanding of the evolution and phylogeography of the Sunda pangolin is crucial for informing species conservation management. Delineating the boundaries of Sunda pangolin populations and/or conservation units, and assessing their genetic diversity, is fundamental to any conservation genetic management efforts (Frankham et al., 2010). Furthermore, the development and application of wildlife forensic tests for this species hinge on sufficient reference data, and on our understanding of its taxonomic boundaries and intraspecific diversity. Forensic applications, such as identifying the species and geographic provenance of seized pangolins and their derivatives, can enhance enforcement of pangolin trafficking crimes, and may provide insights into poaching hotspots, trafficking networks and potential routes of disease transmission along the trade chain. However, to date, pangolin seizures have not always been identified to species level using standard mitochondrial DNA (mtDNA) sequence‐based tests due to low DNA sequence similarity to existing reference sequences (e.g. Zhang et al., 2015). In particular, the National Wildlife Forensic Laboratory in Malaysia has been unable to determine the species of seized pangolin scales for multiple cases due to a lack of comprehensive reference data and the potential presence of cryptic species in the region, hence were unable to determine whether the scales derived from locally poached Sunda pangolin/s, or whether they derived from non‐native pangolin species (i.e. internationally trafficked). In addition, no geographic provenance tests are available, as these rely on substantial geographic sampling and robust phylogeographic information (Ogden & Linacre, 2015), which has not yet been sufficiently developed for this species. Consequently, the geographic origin of Sunda pangolin seizures is often unknown, obfuscating efforts to identify poaching and trafficking patterns, and to manage the potential spill‐over of diseases to humans and other wildlife or domestic species.

Here, we generate 23 Sunda pangolin mtDNA genomes from wild individuals of known provenance sourced from Malaysian Borneo and Peninsular Malaysia. These newly acquired mtDNA genomes were integrated with previously generated mtDNA sequences and nuclear SNP marker data (RAD‐seq data; Nash et al., 2018) to facilitate a range‐wide genetic assessment of the species. These data were utilized in several population genetic and phylogenetic analyses to: (1) characterize the distribution of the Sunda pangolins' genetic diversity, particularly in Borneo, to improve our understanding of the species' phylogeography and evolutionary history, and (2) consider the implications of these findings for the species' taxonomy, conservation genetic management and wildlife forensic applications. Furthermore, we identify key geographic locations where reference samples are still needed, which will help direct further genetic studies on this species.

## 2 METHODS

### 2.1 Sample acquisition and DNA extractions

**FIGURE 1:**
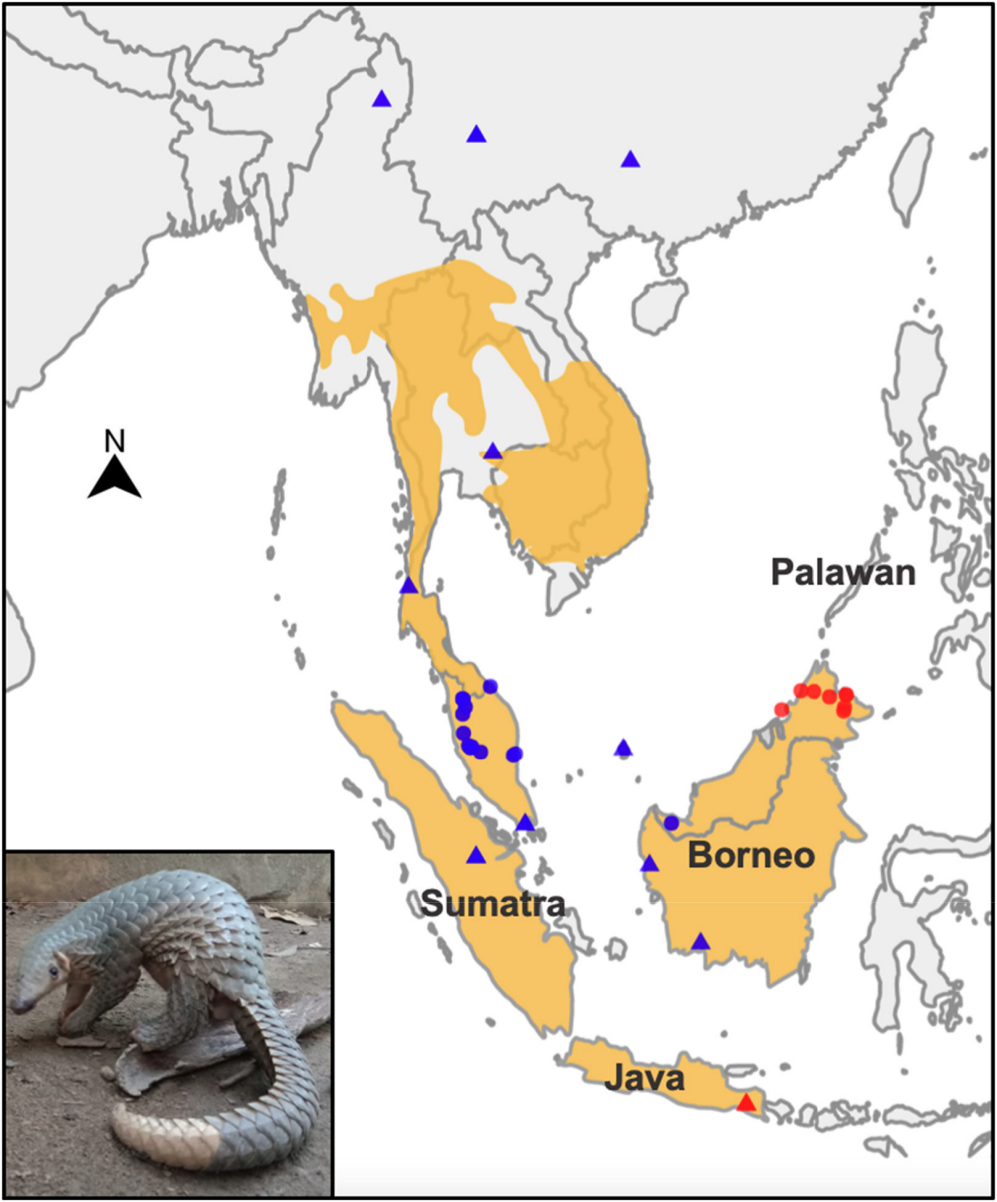
The Sunda pangolin (inset) distribution (orange shading) and localities of reference samples of known origin sequenced (mtDNA) in this study (circles), and reference samples from NCBI (triangles). Points were coloured by their inferred mtDNA clade (see Figure 2). The distribution is based on the IUCN SSC Pangolin Specialist Group website, noting that the northern and western limits of the species distribution are uncertain (Chong et al., 2020). Where coordinates were not available, the sample was plotted in the centre of the recorded region in which they were collected. Samples that failed (Table S1) were not included in this map. The three points north of the shaded distribution are reference samples from Hu, Hao, et al. (2020) and Hu, Roos, et al. (2020) (i.e. the samples from Kachin, Myanmar and Yunnan Province, China) and Gaubert et al. (2018) (i.e. the sample from Guangxi, China). Verifying the provenance of these specimens could extend the known distribution of the Sunda pangolin.

Samples from wild Sunda pangolins were opportunistically collected (e.g. rescues) from Malaysian Borneo (*n* = 11) and Peninsular Malaysia (*n* = 15) between 2018 and 2022 (Figure 1; Table S1). Most of the pangolins were obtained from rescue operations by Malaysian wildlife authorities (i.e. they were injured and/or part of a human–animal conflict case) except for three individuals from Peninsular Malaysia (MJ555, MJ556 and MJ557), which were seized during an enforcement operation (these individuals are believed to be locally sourced), and one individual from north Borneo Sabah (B01a), which was sampled and released during a field survey. In addition, one captive‐born Sunda pangolin (MJ567) was sampled. Blood or hairs were sampled from live pangolins by trained veterinarian officers, and tissues were sampled from deceased individuals (Table S1). Fresh blood samples were drawn using a medical syringe into a blood collection tube containing EDTA to prevent clotting, then stored in a freezer. Tissue samples were stored in absolute ethanol, and hair samples were kept dry in clean zip‐lock bags.

Genomic DNA was extracted from the pangolin samples in Malaysia at the National Wildlife Forensic Laboratory, PERHILITAN, and at the Wildlife Health, Genetic and Forensic Laboratory (WHGFL), Sabah, using the DNeasy Blood and Tissue kit (Qiagen) following the recommended manufacturer's protocol, with a minor modification for hair samples; 20 μL of 1.0 M DTT was added to hair samples during lysis. DNA concentrations were measured using a Qubit Fluorometer (Qubit dsDNA BR Assay).

### 2.2 Generation of mtDNA genomes

To generate full mtDNA genome sequences from the Sunda pangolin DNA samples, we performed low‐coverage ‘genome skimming’ sequencing at Monash University Malaysia Genomics Facility (Selangor, Malaysia). Libraries were prepared following the NEBNext DNA library preparation protocol (New England Biolabs), with a pretreatment of 500 bp shearing using a Covaris M220 focused ultrasonicator (Covaris). Quantification and size estimation of the libraries was performed on a Tapestation 2200 (Agilent) and Qubit fluorometer (Invitrogen). Subsequently, equimolar sample libraries with index adaptors were pooled and sequenced on an Illumina MiSeq desktop sequencer using paired‐end 250 bp sequencing.

The resultant sequence reads were trimmed using BBDuk, implemented in Geneious v.10.2.4 (Kearse et al., 2012), whereby low‐quality ends (quality <6) and adaptor sequences were trimmed, and short reads were discarded (<100 bp). The trimmed sequence data were aligned to numerous Sunda pangolin mtDNA genomes available on NCBI using the Geneious mapper with the medium–low sensitivity setting, iterated up to 10 times. Reads that mapped to any Sunda pangolin mtDNA genome (i.e. reads of putative mtDNA origin) were subsequently utilized in a de novo assembly using the Geneious assembler with the medium sensitivity setting. The consensus sequence from this de novo assembly was subsequently extracted. To verify the mtDNA genome sequence assembled using Geneious, we utilized a second assembly pipeline based on a ‘seed‐extend’ approach, implemented in NOVOPlasty (Dierckxsens et al., 2017). Short regions from the Geneious assembly were used as ‘seeds’ to initiate the NOVOplasty analysis. NOVOplasty uses all sequence reads from the low‐coverage whole genomic sequencing to iteratively extend the seed sequence bidirectionally, until the whole mtDNA genome is assembled. We set the K‐mer at 39 for this seed‐extend process. The two assemblies generated from Geneious and NOVOplasty were aligned using the global alignment algorithms implemented in Geneious, and any ambiguities between assemblies were investigated and amended if necessary. We annotated the assembled genomes by transferring annotations from previously annotated genomes on NCBI using Geneious, and subsequently manually edited the start and ends of annotated genes based on open reading frames. Four samples did not produce adequate data for mtDNA genome assembly and only cytochrome‐b and CO1 could be assembled for one sample (i.e. ‘O01’; Table S1). The same assembly methods were utilized to assemble the raw sequence data from three wild Sunda pangolins sequenced by Hu, Hao, et al. (2020) (Table S1).

### 2.3 Phylogenetic and molecular dating analysis

Phylogenetic relationships and evolutionary timescales were estimated using Bayesian Inference in BEAST v2.6.7 (Bouckaert et al., 2019). First, the assembled mtDNA genomes were aligned with reference mtDNA genomes available on NCBI using global alignment algorithms implemented in Geneious. Only reference genomes from wild pangolins of known origin were included (Gaubert et al., 2018; Hassanin et al., 2015; Hu, Hao, et al., 2020; Mason et al., 2019; Nash et al., 2018; Wirdateti et al., 2022; Table S1); however, the documented origin of some samples may not be precise and/or accurate in some cases (see Figure 1). Second, stop codons and any overlapping regions between genes were removed (as these regions are under complex selective constraints). If this trimming caused sequence alignments to shift out of codon frame, the first one or two nucleotides were removed to ensure the protein‐coding genes were in‐frame. Third, the alignments were partitioned as follows: one partition comprising the 2 ribosomal RNAs (rRNAs) and 22 mitochondrial transfer RNAs (tRNAs), a second partition comprising the first and second codon positions of the 13 protein‐coding genes, a third partition comprising the third codon position of the 13 protein‐coding genes and a fourth partition comprising the control region.

In BEAST, each partition had its own substitution model and clock model. We implemented bModeltest, which enables BEAST to sample different substitution models according to their probabilities. To check the sensitivity of the results to the choice of clock model and tree prior (Ritchie et al., 2017), we implemented both the lognormal relaxed clock and strict clock model, and the birth–death speciation and constant‐size coalescent model in separate analyses. We used two secondary calibrations as priors using estimates from a well‐resolved pangolin phylogeny (Gaubert et al., 2018). The 95% CI Manidae divergence estimates in Gaubert et al. (2018) informed the mean (12.9) and standard deviation (1.65) of a normal prior set for the Manidae ‘time to most recent common ancestor’ (TMRCA), and the mean (9.1) and standard deviation (1.4) of a normal prior set for the divergence between the Indian pangolin (*Manis crassicaudata*) and the Sunda and Palawan pangolin (*Manis culionensis*). Monophyly of the Sunda pangolin and Palawan pangolin was enforced. MCMC was run for 10^8^ steps with a pre‐burn‐in of 10^7^ steps, sampling every 5000 steps. MCMC results were checked in TRACER v1.7.2 (Rambaut et al., 2014) for convergence and sufficient sampling, and a maximum clade credibility tree was generated using Treeannotator v2.6.7 (part of the BEAST package) using median node heights and a 10% burn‐in. Phylogenetic trees were visualized and rooted (using *Manis pentadactyla*) using FigTree v1.4.4 (Rambaut, 2009).

To complement the Bayesian phylogenetic analysis, phylogenetic relationships were estimated among the mtDNA genomes using a maximum likelihood analysis in RAXML v8.0 (Stamatakis, 2014). We implemented the GTR + G substitution model using the same partitioning scheme as in the BEAST analysis, and performed 1000 bootstrap replicates to estimate node support. The maximum likelihood phylogenetic analysis was repeated on a data set comprising concatenated CO1 and cytochrome‐b sequences, and a data set comprising only CO1 sequences, to maximize the Sunda pangolin sampling coverage (Table S1).

### 2.4 Identification of seized Sunda pangolin samples

We used a tree‐based approach to putatively identify the geographic provenance of seized Sunda pangolin samples from previous studies (Gao et al., 2020; Nash et al., 2018; Peng et al., 2021; Zhang et al., 2015) based on the reference data generated in this study. Several samples from previous wildlife forensic cases in Malaysia that were unable to be identified to species level (i.e. they were identified to *Manis* spp.) were also re‐identified. We used RAxML (following the methods outlined above) to construct the phylogenetic trees for these identifications; the gene region(s) analysed depended on the data available from the previous studies.

### 2.5 mtDNA haplotype analyses

We calculated mtDNA divergence between the two major Sunda pangolin populations (detailed in the Sections 3 and 4) and the Palawan pangolin based on mtDNA genomes. Divergence was based on net nucleotide divergence (*Da*), calculated using the R package strataG v.2.4.905 (Archer et al., 2017), and mean pairwise difference, calculated using Geneious.

We performed a haplotype network analysis to further investigate population structure based on mtDNA. We performed this analysis using PopArt (Leigh & Bryant, 2015), implementing the statistical parsimony TCS method (Clement et al., 2000), based on concatenated CO1 and cytochrome‐b sequences to maximize the Sunda pangolin sampling coverage.

Haplotype accumulation curves were constructed with HACSim v1.0.5 (Phillips et al., 2020) to estimate the number of additional Sunda pangolin samples that would be required to recover 80% and 95% of the inferred total number of haplotypes. The HACSim analysis was performed using a 778 bp cytochrome‐b region (i.e. the region in Nash et al., 2018) from wild Sunda pangolins of known origin (*n* = 38; Table S1) combined with sequences from Sunda pangolins of presumed unknown origin from NCBI (*n* = 57). Ambiguous nucleotides were converted to the alignment consensus sites for this analysis. The analysis was performed using all sequences, implementing 10,000 permutations, then repeated for each of the two major Sunda pangolin clades separately to account for the considerable population differentiation (detailed in the Sections 3 and 4). In addition, we identified the number of haplotypes from the samples of unknown origin that did not match any sequences from reference samples of known origin.

### 2.6 SNP analyses

Raw SNP data were ascertained from the Nash et al.'s (2018) ddRADseq study (89 individuals and 60,197 SNPs). We applied numerous SNP filters using the R packages dartR v1.9.9 (Gruber et al., 2018) and poppr v.2.6.1 (Kamvar et al., 2014, 2015). First, SNPs with >20% missing data were removed (23,758 SNPs were retained). Second, individuals containing >25% missing data were removed (83 individuals were retained). Third, to remove potentially artefactual SNPs (e.g. caused by sequencing error or erroneous assembly of paralogs), SNPs with a minor allele frequency < 0.02 (threshold based on 3/2*n*) and an observed heterozygosity >0.8 were removed (8911 SNPs were retained). Fourth, to meet the assumptions of some subsequent population genetic analyses, SNPs were filtered for linkage disequilibrium (LD) and Hardy–Weinberg equilibrium (HWE). Potentially linked SNP with a *r* ^2^ > 0.6, estimated using the R package ‘genetics’ (Warnes, 2003), was pruned, and SNPs that putatively deviated from HWE (*p* < .05, based on 1000 permutations; we considered all samples as one population as a conservative approach), implemented using the R package pegas v0.1 (Paradis, 2010), were removed.

We performed genetic structure analyses on two subsets of samples from Nash et al. (2018). First, to compare nuclear population structure patterns to mtDNA inferences, we retained individuals sourced from the wild and seized individuals that had a corresponding mtDNA haplotype that clearly clustered in one of the three mtDNA clades characterized in this study (see Section 3). Second, to investigate nuclear genetic diversity and population divergence based on a more expansive data set, we extracted individuals sourced from the wild and all seized individuals that clustered into the three distinct populations characterized by Nash et al. (2018), regardless of the corresponding mtDNA inferences. We used three methods to investigate population genetic structure based on these SNP data sets. First, genetic variation was summarized using a principal coordinates analysis (PCoA) performed in R packages dartr and ade4 v.1.7 (Chessel et al., 2004). SNPs were not filtered for HWE or LD for PCoA (all subsequent analyses utilize SNPs filtered for HWE and LD to meet population genetic assumptions). Second, ancestry coefficients were estimated using sparse non‐negative matrix factorization (sNMF), implemented in the R package LEA v3.2 (Frichot & François, 2015; Gain & François, 2021). We modelled up to six ancestral populations (i.e. *K*), replicating each model 10 times. To determine the optimal value of *K* in this analysis, we computed cross‐entropy criterion for each *K* (Frichot et al., 2014). Third, to measure genetic divergence between the populations, we calculated pairwise fixation index (*F*_ST_) values using the R package hierfstat v.0.4.22 (i.e. the Weir & Cockerham, 1984 *F*_ST_ estimate; Goudet, 2005). We computed confidence intervals for the *F*_ST_ values based on 0.025 and 0.975 quantiles, implementing 1000 bootstraps.

Four genetic diversity metrics were estimated based on the SNP data. Observed and expected heterozygosity was estimated using the R package adegenet v 3.5.2 (Jombart, 2008), rarefied allelic richness was assessed using the R package PopGenReport v3.0.4 (Adamack & Gruber, 2014) and private alleles counts were performed using the R package poppr. Alike the pairwise *F*_ST_ analyses, populations were divided based on the SNP clustering analyses.

## 3 RESULTS

### 3.1 Phylogenetic and molecular dating

**FIGURE 2:**
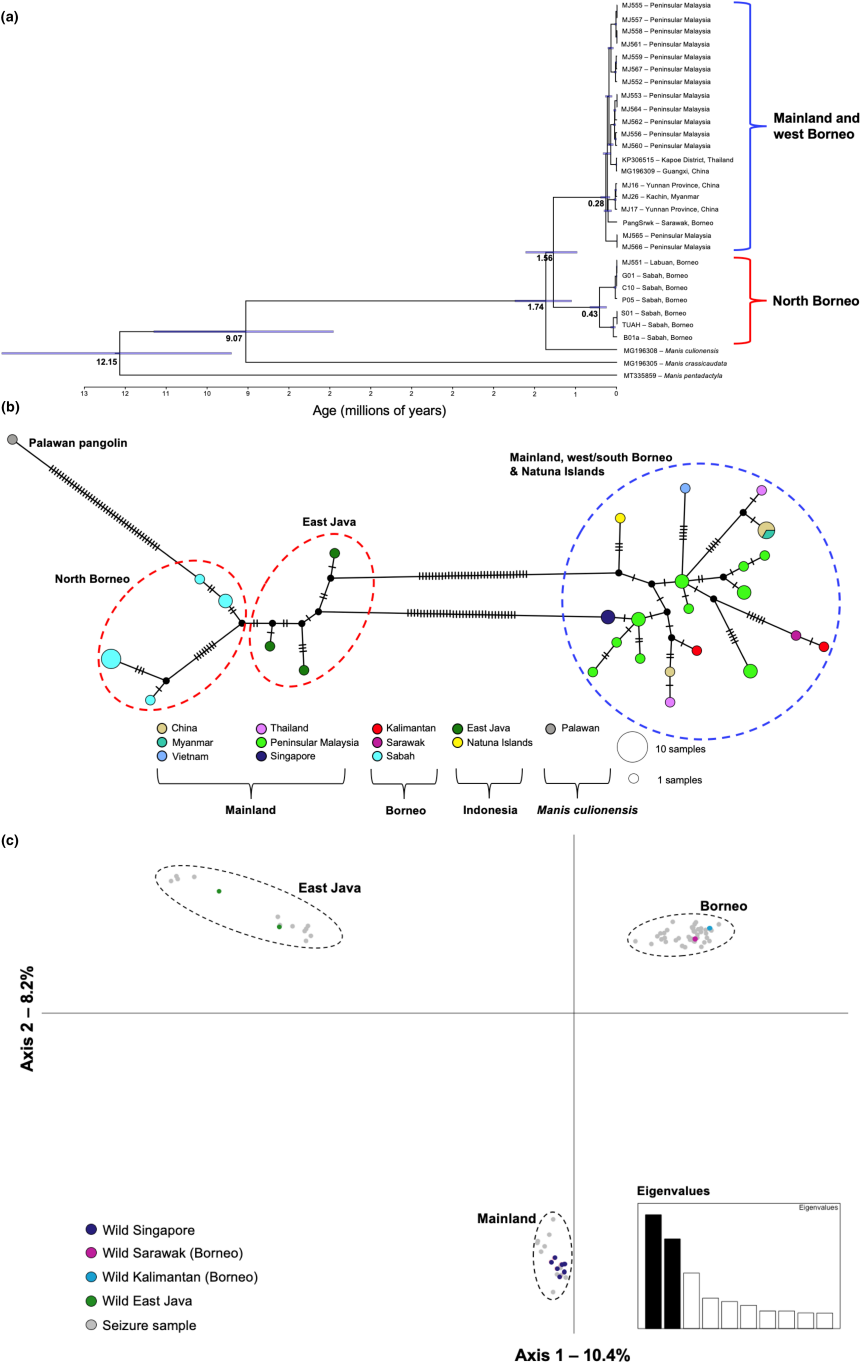
(a) Bayesian molecular dating analysis based on 30 mtDNA genomes (16,387 bp), lognormal relaxed clock model and the birth–death speciation tree prior (results for other clock models and tree priors are presented in Table S2). The age of key nodes are labeled, and blue bars on the tree correspond to the 95% credibility intervals (HPD) of the estimated node ages. (b) TCS‐based haplotype network for 38 Sunda pangolin samples and one Palawan pangolin samples based on 1521 bp of concatenated CO1 and cytochrome‐b; dashes on haplotype network branches represent substitutions, and the sizes of circles are proportional to the number of samples. (c) PCoA plot based on 8853 SNPs and 75 Sunda pangolins from Nash et al. (2018), including wild sourced individuals and all seized individuals that clustered into the three distinct populations characterized by Nash et al. (2018). The colours of the curly braces in ‘a’ and dashed ellipses in ‘b’ correspond to the coloured points in Figure 1.

The phylogenetic analyses revealed three distinct lineages within the Sunda/Palawan pangolin complex (Figure 2a, Figures S1–S3): (1) Sunda pangolins from mainland (from Singapore to China), Sumatra, Natuna Islands and west/south Borneo (i.e. ‘blue’ points in Figure 1); (2) Sunda pangolins from Sabah (i.e. north Borneo) and East Java (i.e. ‘red’ points in Figure 1); and (3) the Palawan pangolin (Philippines). The topology of this species complex differed depending on the phylogenetic method (i.e. maximum likelihood vs. Bayesian inference) and data set analysed (i.e. whole mtDNA genomes and less samples vs. CO1 and cytochrome‐b data with more samples) (Figure 2a, Figures S1–S3). This was particularly germane for the placement of the Palawan pangolin in respect to the different Sunda pangolin clades. There appeared to be some additional structure within the two major Sunda pangolin clades (Figure 2a).

The posterior mean of age of the TMRCA of the Sunda pangolin lineages was 1.54–1.72 mya, depending on the clock and tree models implemented, while the divergence time between the Sunda pangolin and Palawan pangolin was 1.67–1.94 mya (Figure 2a; Table S2). The estimated TMRCA of *Manis* and the TMRCA of the Sunda, Palawan and Indian pangolin falls within the 95% HPD intervals reported by Gaubert et al. (2018). Within north Borneo, there appears to be two divergent mtDNA lineages that diverged 410–510 thousand years ago.

The putative geographic provenance of previously seized Sunda pangolins was inferred via phylogenetic analyses using the newly generated reference data (Table S3). Samples were identified to either the ‘mainland, Sumatra and west/south Borneo’ population, ‘north Borneo’ population or ‘Java’ population. We were unable to infer the origin of the outlier samples from Zhang et al. (2015) as they did not cluster with any samples. Furthermore, the placement of the wild East Java Sunda pangolin samples in some phylogenetic analyses was unclear, obfuscating identifications to this population in some cases (NB: No whole mtDNA genomes were unavailable from the ‘Java’ population).

### 3.2 mtDNA haplotype analyses

The haplotype network analysis revealed comparable patterns to the phylogenetic analyses outlined above (Figure 2b). The highest pairwise mtDNA divergence, based on net nucleotide divergence (*Da*) and mean pairwise distances, was between Sunda pangolins from the ‘north Borneo’ population and the Palawan pangolin, while the lowest divergence was between Sunda pangolins from the ‘mainland and west/south Borneo’ population and Sunda pangolins the ‘north Borneo’ population (Table S4).

Based on haplotype accumulation curves, ~33 and ~165 Sunda pangolin samples are required to recover 80% and 95% of the species' haplotype diversity respectively (Table 1; Figure S4). When performing the analysis for the two major clades separately and combining the results, ~34 and ~152 samples are required to recover 80% and 95% of the Sunda pangolin haplotype diversity respectively (Table 1). In addition, there were 27 haplotypes from pangolins of unknown origin that did not match any reference sequences from pangolins of known origin.

**TABLE 1:** Inferences on the quantity of unsampled haplotype diversity, and the number haplotypes from Sunda pangolins of unknown origin that do not match any reference sequences (of known origin).

Data set analysed	No. of sequences analysed	No. of additional samples required for 80% haplotype recovery	No. of additional samples required for 95% haplotype recovery	No. of haplotypes that do not match any reference sequences of known origin
All samples	95	33	165	27
Samples from ‘mainland, Sumatra and west/south Borneo’ clade	58	22	95	14
Samples from ‘north Borneo and Java’ clade	37	12	57	13

*Note*: The HACsim analyses were based on a 778 bp cytochrome‐b region from both reference samples of known origin (Table S1) and samples of unknown origin.

### 3.3 SNP analyses

The PCoA revealed three major clusters (based on samples of known origin): (1) mainland, (2) Borneo and (3) Java (Figure 2c, Figure S5). The sNMF results supported the three genetic clusters evident in the PCoA (Figures S5C and S6; see Figure S5D for cross‐entropy values). The seizure samples whose mtDNA haplotype clustered within the ‘north Borneo’ and ‘East Java’ mtDNA clades clustered with the Borneo and Java SNP groups respectively (Figure S5). Whereas, seizure samples whose mtDNA haplotype clustered within the ‘mainland and west/south Borneo’ mtDNA clade clustered within either the mainland or Borneo SNP groups, which is indicative of mitonuclear discordance.

For the SNP diversity and *F*_ST_ analyses, seized individuals were assigned to the ‘mainland’, ‘Borneo’ or ‘Java’ populations based on the SNP clustering analyses (Figure 2c, Figure S6). The putative ‘Borneo’ population was the most genetically diverse based on SNPs, followed by the ‘mainland’ population, and then the ‘Java’ population (Table S5). The two lowest pairwise *F*_ST_ estimates were between the ‘Borneo’ population and the other two populations, while the largest pairwise *F*_ST_ estimate was between the ‘mainland’ population and the ‘Java’ population (Table S6). All *F*_ST_ values were considered significant, as their associated confidence intervals did not encompass zero.

## 4 DISCUSSION

We have performed a phylogeographic assessment of one of the world's most highly trafficked but relatively understudied mammals, the Sunda pangolin. Our analyses of mtDNA genomes in conjunction with previously generated mtDNA and SNP data revealed considerable intraspecific diversity within the Sunda pangolin, and a distinct evolutionary lineage in the north Borneo region. These new phylogeographic inferences will support Sunda pangolin conservation genetic management, and the design and interpretation of wildlife forensic testing involving this species.

### 4.1 Phylogeographic inference

All mtDNA analyses exhibited a split between a ‘north Borneo and Java’ group and a ‘mainland, Sumatra and west/south Borneo’ group (‘mainland’ includes pangolins from Singapore, Peninsular Malaysia, Thailand, Vietnam, Myanmar and China; Figure 1). These two distinct mtDNA lineages diverged from one another ~1.56 mya during the Pleistocene (Figure 2a). Conversely, all individuals derived from Borneo cluster together based on analyses of nuclear SNPs (Figure 2c, Figures S5 and S6), including one individual from Sarawak and one from Kalimantan, indicating the presence of mitonuclear discordance. This putative ‘Borneo’ SNP cluster was the most genetically diverse (Table S5), and was more genetically similar to the ‘mainland’ and ‘Java’ SNP clusters than ‘mainland’ and ‘Java’ were to each other (Table S6).

Taken together, these inferences are consistent with the ‘out of Borneo’ hypothesis proposed by Mason et al. (2019): Sunda pangolins originated in Borneo, evolving multiple mtDNA lineages before subsequently migrating to southern Philippines, the Southeast Asian mainland and Sumatra, and then to Java. Accordingly, the lower SNP diversity in the ‘mainland’ and ‘Java’ populations could be due to the founder effect after colonization from Borneo. Dispersal to different land masses could have been enabled by the major geological changes of Sundaland throughout the Miocene and Pleistocene. In the early Miocene (~5 mya), the Sunda shelf was mostly connected above sea level (i.e. Peninsular Malaysia, Borneo, Sumatra and Java). Subsequently, in the late Miocene and throughout the Pleistocene, glacial cycles caused sea‐level fluctuations with repeated emergence and submergence of land bridges between isolated landmasses. Based on the molecular dating estimates (Figure 2a; Table S2), this dispersal between Borneo, Java, Palawan and the mainland likely occurred within the last 2 million years via exposed land masses, with potential periods of subsequent introgression. In the context of Manidae evolution, Gaubert et al. (2018) indicated that the Asian and African pangolins’ lineages diverged before the Oligocene–Miocene boundary (~22.9 mya), and the Asian pangolins subsequently diversified from ~12.15 mya (Figure 2a). After its divergence from the Indian pangolin ~9.07 mya, the Sunda/Palawan pangolin lineage may have become restricted to Borneo's highland refugia during sea level and climate fluctuations since the Miocene (Haq et al., 1987). Regions of western and northern Borneo remained subaerial throughout the Cenozoic (Moss & Wilson, 1998) and are home to many evolutionary distinct mammal species endemic to Borneo (Hawkins et al., 2016).

Following the ‘out of Borneo’ hypothesis, the mtDNA divergence that presumably accumulated within Borneo may be the result of isolation by vicariance occurring across mountains separating Sabah from Sarawak and Kalimantan, a common biogeographic barrier, or through the formation of rivers across central/northern Borneo such as the Rajang River, or isolation of multiple Pleistocene rainforest refugia (Gorog et al., 2004; Leonard et al., 2015; Mason et al., 2019). The east–west Borneo mtDNA divergence we identified (Figure 1) resembles the phylogeography of many bird species and some mammal species, such as the Sunda colugo (*Galeopterus variegatus*; Mason et al., 2019), lesser mouse deer (*Tragulus kanchil*; Mason et al., 2019), red spiny rat (*Maxomys surifer;* Gorog et al., 2004), oriental magpie‐robin (*Copsychus saularis*; Sheldon et al., 2009) and the mountain black‐eye (*Chlorocharis emiliae*; Gawin et al., 2014). This relatively high mtDNA genetic differentiation within Borneo, and the negligible mtDNA differentiation between west/south Borneo and the mainland, suggests the mainland was colonized relatively recently by populations from west/south Borneo and has diverged in allopatry (leading to some differentiation at nuclear markers). The Borneo, mainland and Java SNP clusters (Nash et al., 2018) may therefore reflect relatively recent divergence between these populations, while the mtDNA differentiation may be the result of older divergence within Borneo. Any barriers to gene flow within Borneo do not appear to be affecting contemporary Sunda pangolin populations given the lack of nuclear DNA differentiation detected across Borneo (though more reference data are required to fully investigate nuclear DNA differentiation within Borneo). The lack of mtDNA genetic structure exhibited between Sunda pangolins from mainland and Sumatra (and Natuna Islands; Figure 2b) is consistent with the phylogeographic patterns of many Sundaland taxa, and reflects the presence of relatively recent forest habitats joining these regions across an exposed continental shelf (Leonard et al., 2015).

An alternative scenario to this ‘out of Borneo’ hypothesis involves ancestral (~1.56 mya) allopatric divergence between the mainland and Borneo (i.e. the Sunda pangolin did not necessarily originate in Borneo), subsequent secondary contact between the mainland and Borneo, followed by more recent allopatric divergence after the submergence of land bridges. During this secondary contact, nuclear gene flow may have homogenized genetic differentiation within Borneo much more rapidly than mtDNA, particularly if male‐biased dispersal occurred (Prugnolle & de Meeus, 2002). Hence, the apparent mtDNA structure we have detected may reflect a partial replacement of mtDNA haplotypes in west/south Borneo during the secondary contact. This could result in a cline of haplotype frequencies occurring across Borneo (i.e. frequent ‘mainland’ haplotypes in western Borneo, which become increasingly rare towards north Borneo). Given that Borneo comprises the highest nuclear genetic diversity (Table S5), this secondary contact might have involved asymmetrical gene flow, whereby individuals from mainland more readily dispersed into Borneo, increasing admixture in Borneo. Following secondary contact between the mainland and Borneo and the colonization of Java, allopatric divergence between the landmasses would have resulted in the contemporary ‘Borneo’, ‘mainland’ and ‘Java’ clusters exhibited in the nuclear SNP data. This secondary contact scenario and the ‘out of Borneo’ hypothesis are not mutually exclusive. Nash et al. (2018) indicated a different secondary contact scenario, whereby introgression occurred from the mainland to Java, and from Borneo to Java. Teasing apart the possible evolutionary scenarios of the Sunda pangolin and Palawan pangolin requires a more comprehensive data set (discussed further below).

### 4.2 Taxonomic and conservation genetic implications

The Sunda and Palawan pangolin diverged ~1.74 mya based on molecular dating (Figure 2a). This timing supports the hypothesis that the Palawan pangolin derived from Borneo via early Pleistocene land bridges across the Greater Palawan shelf, and subsequently became isolated through sea level rises (Gaubert & Antunes, 2005). However, the phylogenetic position of the Palawan pangolin in respect to the Sunda pangolin lineages is not well resolved, possibly due to the relatively short‐spaced divergence events between the clades (i.e. between ~1.56 and ~2.74 mya) and/or the paucity of reference samples available for this species. The Palawan pangolin was previously often considered a subspecies of the Sunda pangolin until a morphological assessment in 2005 identified several skull and scale characters supporting its elevation as a separate species (Gaubert & Antunes, 2005). Genetic analyses of additional Palawan pangolin samples based on both mtDNA and nuclear DNA markers are required to clarify its evolutionary relationship with the Sunda pangolin.

We found some evidence suggesting that the divergent north Borneo and Java lineages could be considered a separate species or subspecies. The Palawan pangolin, the ‘mainland, Sumatra and west/south Borneo’ Sunda pangolin lineage and the ‘north Borneo’ Sunda pangolin lineage are approximately equidistant based on nucleotide divergence (Table S4); however, nuclear data suggest a single contemporary Borneo population (Figure 2c, Figures S5 and S6). In addition, Hu, Roos, et al. (2020) suggested the existence of another Sunda pangolin species of unknown origin based on outlier haplotypes from seizure samples (seized in Hong Kong; Zhang et al., 2015). These outlier haplotypes have been identified multiple times in subsequent law enforcement case work in Malaysia (unpublished data), and exhibit low sequence similarities to any reference sequences analysed in this study (i.e. the highest sequence similarity to the reference sequences was 91.7% and 95.5% based on 600 bp of CO1 and 399 bp of cytochrome‐b respectively). Clarifying these taxonomic uncertainties and resolving the Sunda/Palawan pangolin complex is essential to underpin conservation genetic strategies that maximize the species' evolutionary potential.

Apportioning within‐species genetic variation into conservation units is a fundamental goal of conservation genetics (Frankham et al., 2017; Moritz, 1994). Nash et al. (2018) identified three putative Sunda pangolin conservation units (while indicating that further research was required to support these findings): Borneo, Java and Singapore/Sumatra. Our data do not refute these delineations; however, we have demonstrated that a considerable quantity of intraspecific variation likely remains unsampled for this species, evidenced by the haplotype accumulation curve analyses (Table 1). Thus, although we have contributed a considerable number of reference sequences in this study, additional georeferenced samples are required to adequately characterize the distribution of genetic variation to ensure effective conservation genetic management. These sampling efforts should focus on Borneo, given the considerable mtDNA structure we identified across the island, as well as Indonesia and Palawan. There are no sequences from wild pangolins of known provenance available from east or north Kalimantan, and very few available from Sarawak, west and central Kalimantan, Java and Palawan. In addition, very little nuclear data have been produced from wild Sunda and Palawan pangolins (Gaubert et al., 2018; Nash et al., 2018). Although characterizing conservation units is typically based on mtDNA variation (Moritz, 1994), given the apparent mitonuclear discordance we have identified, both mtDNA and nuclear data should be generated from the georeferenced samples and integrated into analyses apportioning intraspecific genetic variation (e.g. Ewart et al., 2020).

### 4.3 Wildlife forensic implications

The data produced in this study along with our novel phylogeographic inferences provide critical baseline information for wildlife forensic testing involving the Sunda pangolin. These data have helped refine species identification testing for this species complex (e.g. robustly differentiating the Sunda pangolin and Palawan pangolin), and may support the development of traceability tests (Nash et al., 2018). Geographic provenance testing based on mtDNA is likely only feasible for some Sunda pangolin populations. Distinguishing individuals from parts of Borneo and the mainland may be problematic due to the lack of reciprocal monophyly at mtDNA loci; however, deducing geographic provenance for the north Borneo and Java clades may be possible. For example, if a seized pangolin exhibit was tested using a standardized mtDNA marker appropriate for species identification (e.g. a 307 bp cytochrome‐b region; Ewart et al., 2021), and the ‘north Borneo’ haplotype sequence was retrieved, one could exclude the mainland and Sumatra as the geographic provenance of the sample; a longer gene region/s could then be utilized to elucidate whether the sample derived from Borneo or Java (e.g. Figure 2b, Figure S2). Inferring the origin of seized pangolins provides valuable intelligence for trafficking investigations, and when co‐analysed with virus screening, will support the monitoring and management of the potential risk of disease spill‐over events (Lee et al., 2020).

We were able to utilize the new reference data produced in this study to clarify species identity and infer the provenance of previously seized pangolin specimens (Table S3). Several forensic casework samples in Malaysia that were originally reported to *Manis* spp. due to the lack of genetically similar reference samples were able to be identified as Sunda pangolins. Furthermore, we inferred the putative origin of three diseased pangolins seized in China (seized in Lishui, Dongyang and Wucheng; Gao et al., 2020) that were found to be infected with novel RNA viruses. Gao et al. (2020) used seized individuals as reference samples to determine the origin of these pangolins (and assumed that the seizure location of these references corresponded to their provenance) and deduced that they derived from Indonesia, Malaysia and Thailand. However, based on phylogenetic analyses using data produced in our study, they likely derived from north Borneo (MN365836), and the ‘mainland, Sumatra and west/south Borneo’ population (MN365833 & MN365835).

## 5 CONCLUSION

Studies characterizing the phylogeography of the Sunda pangolin have been hampered by limited Sunda pangolin reference samples of known provenance. We have helped to address this by sequencing an additional 23 reference samples from Borneo and Peninsular Malaysia. These data have elucidated the evolution and phylogeographic history of the Sunda pangolin. The identification of a genetically distinct Sunda pangolin population in Borneo could have significant taxonomic and conservation genetic implications. Furthermore, the identification of considerable genetic structure within the species provides important evolutionary context for DNA‐based species identification testing of Asian pangolin seizures, and could enable geographic provenance testing for trafficked Sunda pangolin specimens, an important tool for mitigating the illegal trade of the species and associated disease risks.

## AUTHOR CONTRIBUTIONS

**Frankie T. Sitam:** Conceptualization (lead); data curation (equal); formal analysis (equal); funding acquisition (equal); resources (equal); visualization (equal); writing – original draft (equal); writing – review and editing (equal). **Milena Salgado‐Lynn:** Funding acquisition (equal); resources (equal); writing – review and editing (equal). **Azroie Denel:** Resources (equal); writing – review and editing (equal). **Elisa Panjang:** Resources (equal); writing – review and editing (equal). **Ross McEwing:** Conceptualization (equal); funding acquisition (equal); writing – review and editing (equal). **Amanda Lightson:** Data curation (equal); formal analysis (equal); visualization (equal); writing – review and editing (equal). **Rob Ogden:** Formal analysis (equal); funding acquisition (equal); writing – review and editing (equal). **Nur Alwanie Maruji:** Data curation (equal); resources (equal); writing – review and editing (equal). **Nurhartini Kamalia Yahya:** Data curation (equal); resources (equal); writing – review and editing (equal). **Cosmas Ngau:** Resources (equal); writing – review and editing (equal). **Noor Azleen Mohd Kulaimi:** Resources (equal); writing – review and editing (equal). **Hartini Ithnin:** Resources (equal); writing – review and editing (equal). **Jeffrine Rovie‐Ryan:** Funding acquisition (equal); resources (equal); writing – review and editing (equal). **Mohd Soffian Abu Bakar:** Resources (equal); writing – review and editing (equal). **Kyle M. Ewart:** Conceptualization (equal); formal analysis (lead); visualization (equal); writing – original draft (lead); writing – review and editing (equal).

## Supporting information


Appendix S1

## Data Availability

All mitochondrial DNA sequence data generated in this study are available in GenBank (accession numbers OR327007‐OR327028, OR343193 & OR347008). Metadata associated with the samples are available in the Appendix S1.
