# Supplementary material for: Phylogeography of the Sunda pangolin, Manis javanica: Implications for taxonomy, conservation management and wildlife forensics
Source: Ecol Evol. 2023 Aug 15;13(8):e10373. doi: 10.1002/ece3.10373 (PMC10427774; doi:10.1002/ece3.10373)
Supplement: Supplementary file 1 — Appendix S1 [file ECE3-13-e10373-s001.docx]

**Supplementary Material**

**Table S1.** Details of the Sunda pangolin sequenced in this study. The samples derived from the National Wildlife Forensic Laboratory (Perhilitan), Malaysia, and the Danau Girang Field Centre (DGFC), Sabah, Malaysia.

| **Sample name** | **Wild/Captive** | **Collection** | **Geographic provenance** | **Date collected** | **Sample type** | **Study** | **mtDNA region analysed** |
| --- | --- | --- | --- | --- | --- | --- | --- |
| B01a | Wild | DGFC | Lower Kinabatangan Wildlife Sanctuary, Sabah | 23/3/2019 | Blood | Current study | mtDNA genome |
| P05 | Wild (confiscated live pangolin of known provenance) | DGFC | Sandakan, Sabah | 11/10/2018 | Blood | Current study | mtDNA genome |
| G01 | Wild | DGFC | Ranau, Sabah | 18/9/2019 | Blood | Current study | mtDNA genome |
| Tuah | Wild | DGFC | Sandakan, Sabah | 21/10/2018 | Hair | Current study | mtDNA genome |
| Wira | Wild | DGFC | Batu 12, Sandakan, Sabah | 3/8/2018 | Hair | Current study | Data insufficient for mtDNA assembly |
| P01 | Wild | DGFC | Borneo Samudera Plantation, Kinabatangan, Sabah | 9/12/2016 | Blood | Current study | Data insufficient for mtDNA assembly |
| O01 | Wild | DGFC | Beluran, Sabah | 15/2/2020 | Blood | Current study | CO1 and cytochrome-b |
| S01 | Wild | DGFC | Plantation near Kampung Paris, Kinabatangam, Sabah | 9/4/2020 | Blood | Current study | mtDNA genome |
| C10 | Wild (confiscated live pangolin of known provenance) | DGFC | Kota Kinabalu, Sabah | 29/3/2017 | Hair | Current study | mtDNA genome |
| MJ555 | Wild (confiscated live pangolin of known provenance) | Perhilitan | Pengkalan Hulu, Perak, Peninsular Malaysia | 24/9/2019 | Tissue | Current study | mtDNA genome |
| MJ556 | Wild (confiscated live pangolin of known provenance) | Perhilitan | Pengkalan Hulu, Perak, Peninsular Malaysia | 24/9/2019 | Tissue | Current study | mtDNA genome |
| MJ557 | Wild (confiscated live pangolin of known provenance) | Perhilitan | Pengkalan Hulu, Perak, Peninsular Malaysia | 24/9/2019 | Tissue | Current study | mtDNA genome |
| MJ558 | Wild | Perhilitan | Lahat, Ipoh, Perak, Peninsular Malaysia | 08/07/2020 | Blood | Current study | mtDNA genome |
| MJ559 | Wild | Perhilitan | Kota Bharu, Kelantan, Peninsular Malaysia | 08/07/2020 | Blood | Current study | mtDNA genome |
| MJ560 | Wild | Perhilitan | Gerik, Perak, Peninsular Malaysia | 08/07/2020 | Blood | Current study | mtDNA genome |
| MJ561 | Wild | Perhilitan | Bidor, Perak, Peninsular Malaysia | 08/07/2020 | Blood | Current study | mtDNA genome |
| MJ562 | Wild | Perhilitan | Teluk Cempedak, Kuantan, Pahang, Peninsular Malaysia | 22/07/2020 | Tissue | Current study | mtDNA genome |
| MJ564 | Wild | Perhilitan | Lenggong, Perak, Peninsular Malaysia | 22/07/2020 | Tissue | Current study | mtDNA genome |
| MJ565 | Wild | Perhilitan | PKHL Sungkai, Perak, Peninsular Malaysia (offspring of MJ566) | 22/07/2020 | Tissue | Current study | mtDNA genome |
| MJ566 | Wild | Perhilitan | Kg. Jernang, Sungkai, Perak, Peninsular Malaysia | 11/06/2020 | Tissue | Current study | mtDNA genome |
| MJ567 | Captive | Perhilitan | PKHL Sungkai, Perak, Peninsular Malaysia | 17/08/2020 | Tissue | Current study | mtDNA genome |
| MJ551 | Wild | Perhilitan | Labuan, Sabah | 05/07/2018 | Tissue | Current study | mtDNA genome |
| MJ552 | Wild | Perhilitan | Raub, Pahang, Peninsular Malaysia | 29/09/2019 | Tissue | Current study | mtDNA genome |
| MJ553 | Wild | Perhilitan | Kuantan, Pahang, Peninsular Malaysia | 28/06/2019 | Hair | Current study | mtDNA genome |
| Tomas | Wild | Perhilitan | Perak, Peninsular Malaysia | 23/10/2022 | Blood | Current study | Data insufficient for mtDNA assembly |
| Menderang | Wild | Perhilitan | Menderang, Sungkai, Perak, Peninsular Malaysia | 03/02/2022 | Blood | Current study | Data insufficient for mtDNA assembly |
| PangSrwk | Wild | Perhilitan | Padawan, Kuching, Sarawak | 17/05/2022 | Tissue | Current study | mtDNA genome |
| KP306515 | Wild | GenBank | Kapoe District, Thailand | - | - | Hassanin *et al*., (2015) | mtDNA genome |
| MG196309 | Wild | GenBank | Guangxi, China | - | - | Gaubert *et al.* (2018) | mtDNA genome |
| MJ16 (SRR9018664) | Wild | GenBank | Yunnan Province, China | - | - | Hu *et al*. (2020) | mtDNA genome |
| MJ17 (SRR9018665) | Wild | GenBank | Yunnan Province, China | - | - | Hu *et al*. (2020) | mtDNA genome |
| MJ26 (SRR9018633) | Wild | GenBank | Kachin, Myanmar | - | - | Hu *et al*. (2020) | mtDNA genome |
| MZBR_  1179 | Wild | GenBank | Jember, East Java, Indonesia | - | - | Nash *et al.* (2018) | CO1 and cytochrome-b |
| MZBR_  1180 | Wild | GenBank | Jember, East Java, Indonesia | - | - | Nash *et al.* (2018) | CO1 and cytochrome-b |
| MZBR_  1184 | Wild | GenBank | Jember, East Java, Indonesia | - | - | Nash *et al.* (2018) | CO1 and cytochrome-b |
| MZBR_  1163 | Wild | GenBank | Pangkalan Bun, Central Kalimantan, Indonesia | - | - | Nash *et al.* (2018) | CO1 and cytochrome-b |
| Singapore_  1 | Wild | GenBank | Singapore | - | - | Nash *et al.* (2018) | CO1 and cytochrome-b |
| Singapore_  8b | Wild | GenBank | Singapore | - | - | Nash *et al.* (2018) | CO1 and cytochrome-b |
| 356430 | Wild | GenBank | Mount Santra Area, Vietnam | - | - | Mason *et al.* (2019) | CO1 and cytochrome-b |
| 260592 | Wild | GenBank | Ban Ku Khano, Thailand | - | - | Mason *et al.* (2019) | CO1 and cytochrome-b |
| 142460 | Wild | GenBank | Pontianak, West Kalimantan, Indonesia | - | - | Mason *et al.* (2019) | CO1 and cytochrome-b |
| 104598 | Wild | GenBank | Natuna Islands (Bunguran), Indonesia | - | - | Mason *et al.* (2019) | CO1 and cytochrome-b |
| MZBR1123 | Wild | Wirdateti *et al.* (2022) | Riau, Sumatra, Indonesia | - | - | Wirdateti *et al.* (2022) | CO1 |
| MZBR1124 | Wild | Wirdateti *et al.* (2022) | Riau, Sumatra, Indonesia | - | - | Wirdateti *et al.* (2022) | CO1 |
| MG196308 | - | GenBank | Palawan pangolin (*Manis culionensis*) | - | - | Gaubert *et al.* (2018) | mtDNA genome |
| MT335859 | - | GenBank | Chinese pangolin (*Manis pentadactyla*) | - | - | Hua *et al.* (2020) | mtDNA genome |
| MG196305 | - | GenBank | Indian pangolin (*Manis crassicaudata*) | - | - | Gaubert *et al.* (2018) | mtDNA genome |

**Table S2.** Estimated times to most recent common ancestor (MRCA) for key nodes based on analyses of mtDNA genomes in BEAST2 analyses, implementing four different model combinations. Secondary calibrations were defined on nodes marked with an asterisk.

| **Node** | **Lognormal relaxed clock; birth-death speciation tree prior** | | **Strict clock; birth-death speciation tree prior** | | **Lognormal relaxed clock; constant-size coalescent tree prior** | | **Strict clock; constant-size coalescent tree prior** | |
| --- | --- | --- | --- | --- | --- | --- | --- | --- |
|  | **Median age** | **95% HPD** | **Median age** | **95% HPD** | **Median age** | **95% HPD** | **Median age** | **95% HPD** |
| *Manis* MRCA* | 12.15 | 9.42 - 15.03 | 12.14 | 9.71 - 14.67 | 12.46 | 9.70 - 15.32 | 12.29 | 9.79 - 14.72 |
| *M. crassicaudata -* (*M. javanica, M. culionensis*) MRCA* | 9.07 | 6.93 - 11.32 | 9.16 | 7.28 - 11.02 | 8.99 | 6.88 - 11.40 | 9.25 | 7.33 - 11.07 |
| *M. javanica - M. culionensis* MRCA | 1.74 | 1.11 - 2.49 | 1.67 | 1.31 - 2.07 | 1.94 | 1.22 - 2.91 | 1.71 | 1.32 - 2.10 |
| *M. javanica* MRCA | 1.56 | 0.98 - 2.23 | 1.54 | 1.20 - 1.89 | 1.72 | 1.05 - 2.53 | 1.58 | 1.23 - 1.95 |
| *M. javanica* ‘north Borneo’ clade MRCA | 0.43 | 0.26 - 0.66 | 0.41 | 0.30 - 0.52 | 0.51 | 0.29 - 0.87 | 0.42 | 0.31 - 0.54 |
| *M. javanica* ‘mainland and west Borneo’ clade MRCA | 0.28 | 0.18 - 0.40 | 0.26 | 0.19 - 0.33 | 0.33 | 0.20 - 0.55 | 0.27 | 0.20 - 0.34 |


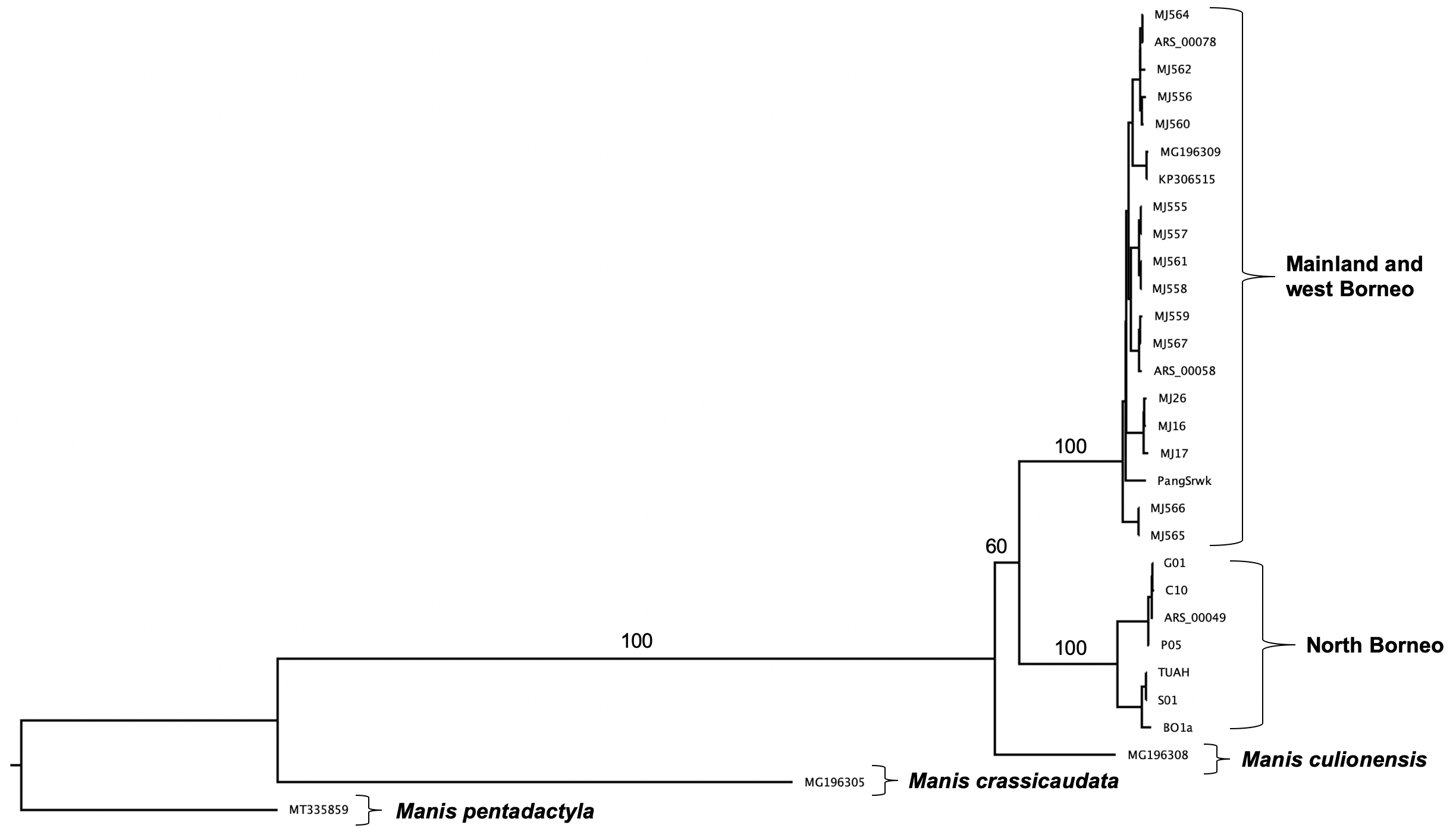


**Figure S1.** Maximum likelihood phylogenetic analysis based on 30 samples and full mtDNA genomes (16,387 bp), divided into 4 partitions, and implementing the GTR+G model. Bootstrap support (%) is given above the branches.


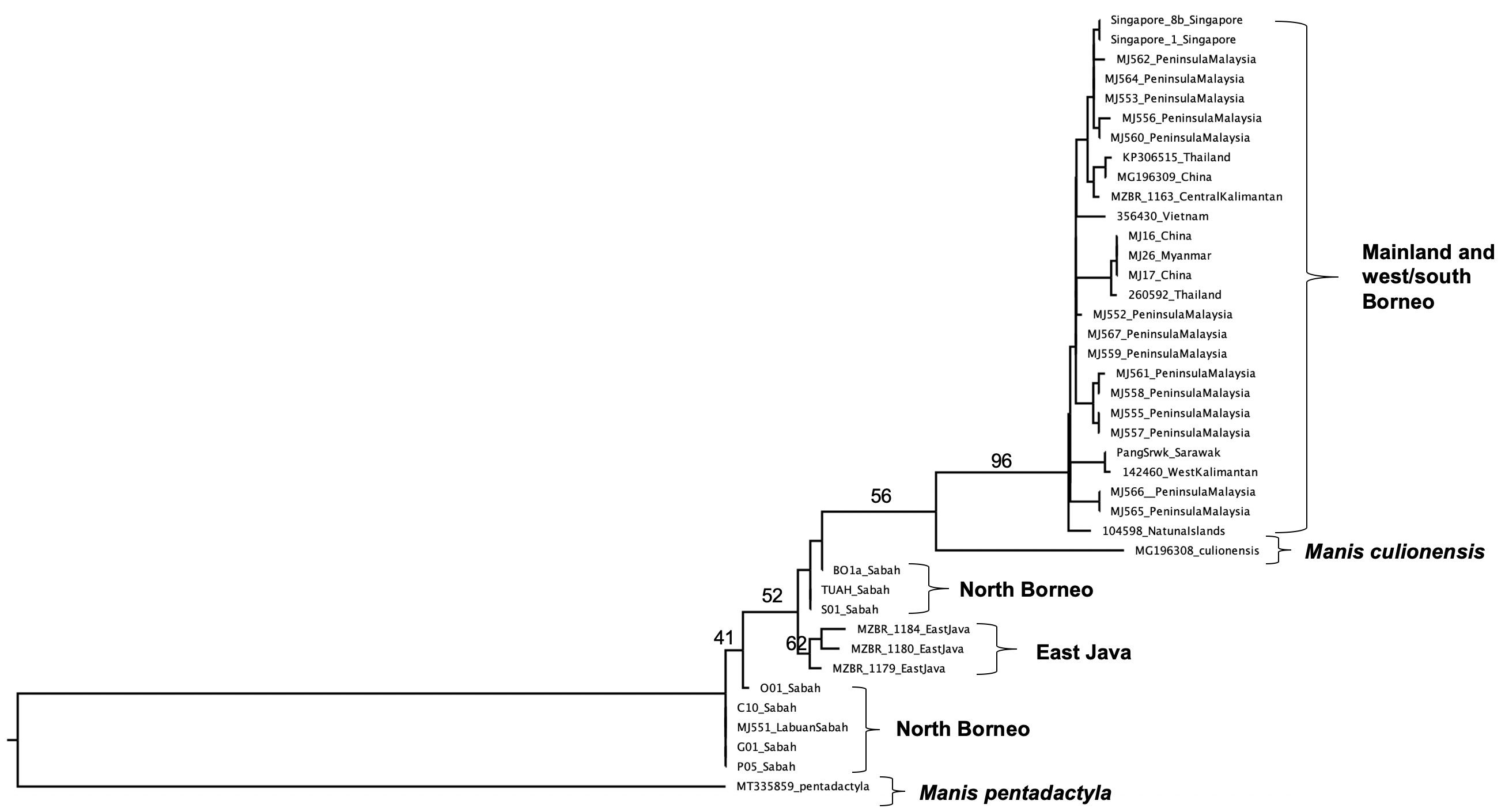


**Figure S2.** Maximum likelihood phylogenetic analysis based on 40 samples and 1,518 bp of concatenated cytochrome-b and CO1 sequences, divided into 2 partitions, and implementing the GTR+G model. Bootstrap support (%) is given above key branches.

**
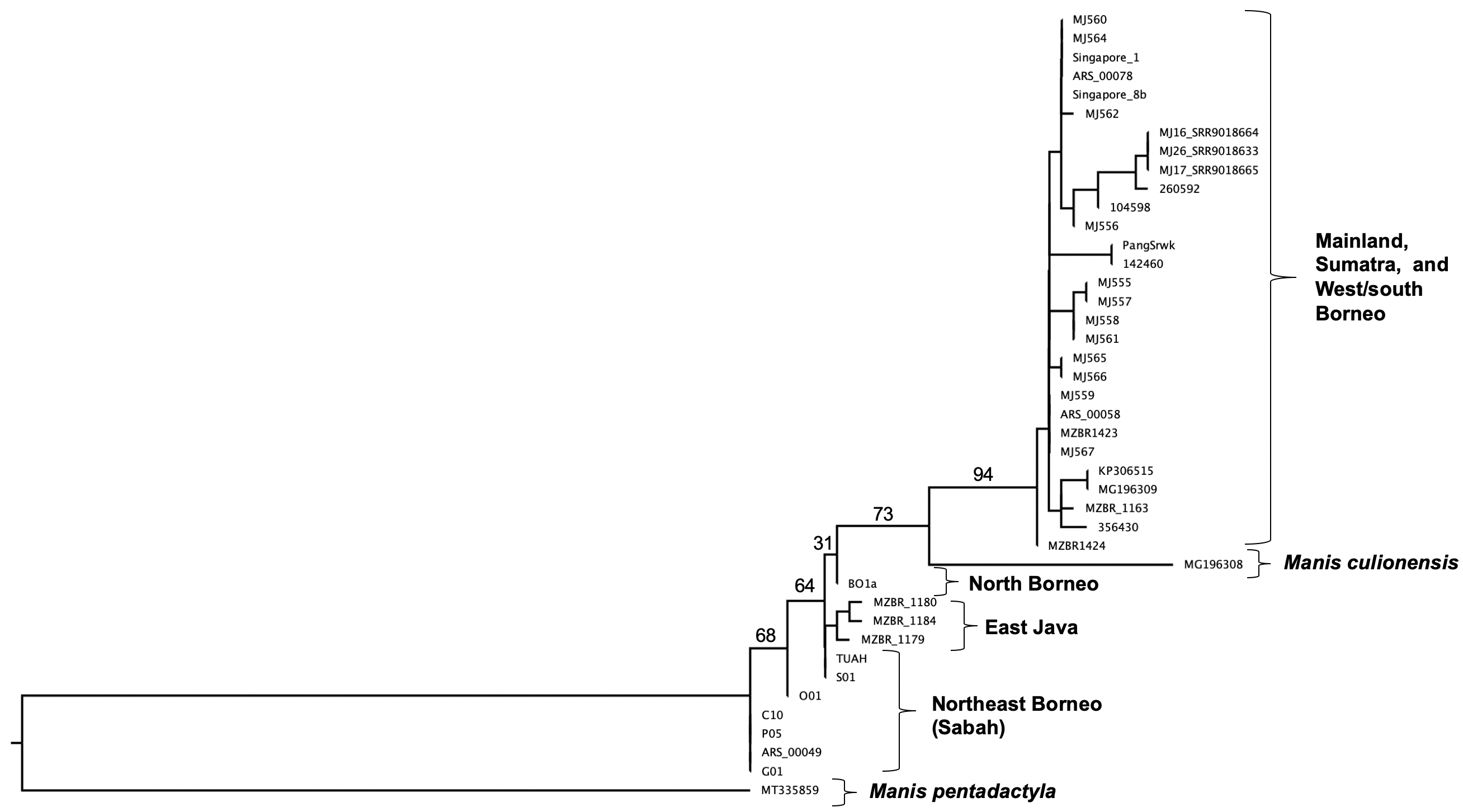
**

**Figure S3.** Maximum likelihood phylogenetic analysis based on 42 samples and 738 bp of CO1 sequences, divided into 4 partitions, and implementing the GTR+G model. Bootstrap support (%) is given above key branches.

**Table S3.** Putative geographic origin of previously seized Sunda pangolins.

| **Sample name** | **Sample description** | **Study** | **Gene region/s available** | **Previous putative provenance** | **Updated putative provenance** |
| --- | --- | --- | --- | --- | --- |
| 1-Dongyang | Diseased pangolin seized in China | Gao et al. (2020) | Cyt-b | Malaysia | Mainland, Sumatra, or west/south Borneo |
| 2-Lishui | Diseased pangolin seized in China | Gao et al. (2020) | mtDNA genome | Indonesia | North Borneo |
| 4-Wucheng | Diseased pangolin seized in China | Gao et al. (2020) | Cyt-b | Thailand | Mainland, Sumatra, or west/south Borneo |
| MZBR_1166 | Seizure in Pangkalanbun, Central Kalimantan | Nash et al. (2018) | Genome-wide SNP, cyt-b & CO1 | Borneo | North Borneo |
| MZBR_1177 | Seizure in Lampung, Sumatra | Nash et al. (2018) | Cyt-b & CO1 | Not assigned; mtDNA clusters with Borneo samples. | North Borneo |
| MZBR_1178 | Seizure in Lampung, Sumatra | Nash et al. (2018) | Cyt-b & CO1 | Not assigned; mtDNA clusters with Borneo. | North Borneo |
| MJ18007 | Diseased pangolin seized in the Yunnan Province, China | Peng et al. (2021) | Cyt-b & CO1 | SE Asia islands, excluding Java | Mainland, Sumatra, or west/south Borneo |
| H4 | Seizure in Hong Kong | Zhang et al., 2015 | Cyt-b & CO1 | Unknown | Unknown |
| H8 | Seizure in Hong Kong | Zhang et al., 2015 | Cyt-b & CO1 | Unknown | Unknown |

**Table S4.** Pairwise mtDNA divergence (using 16,372 bp) based on net nucleotide divergence (*Da*), below the diagonal, and mean pairwise difference, above the diagonal (both as a percentage).

| **mtDNA clade** | **(1)** | **(2)** | **(3)** |
| --- | --- | --- | --- |
| **(1)** Palawan pangolin | - | 3.02 | 3.09 |
| **(2)** Sunda pangolin - mainland & west/south Borneo | 2.80 | - | 2.92 |
| **(3)** Sunda pangolin - north Borneo | 2.82 | 2.43 | - |


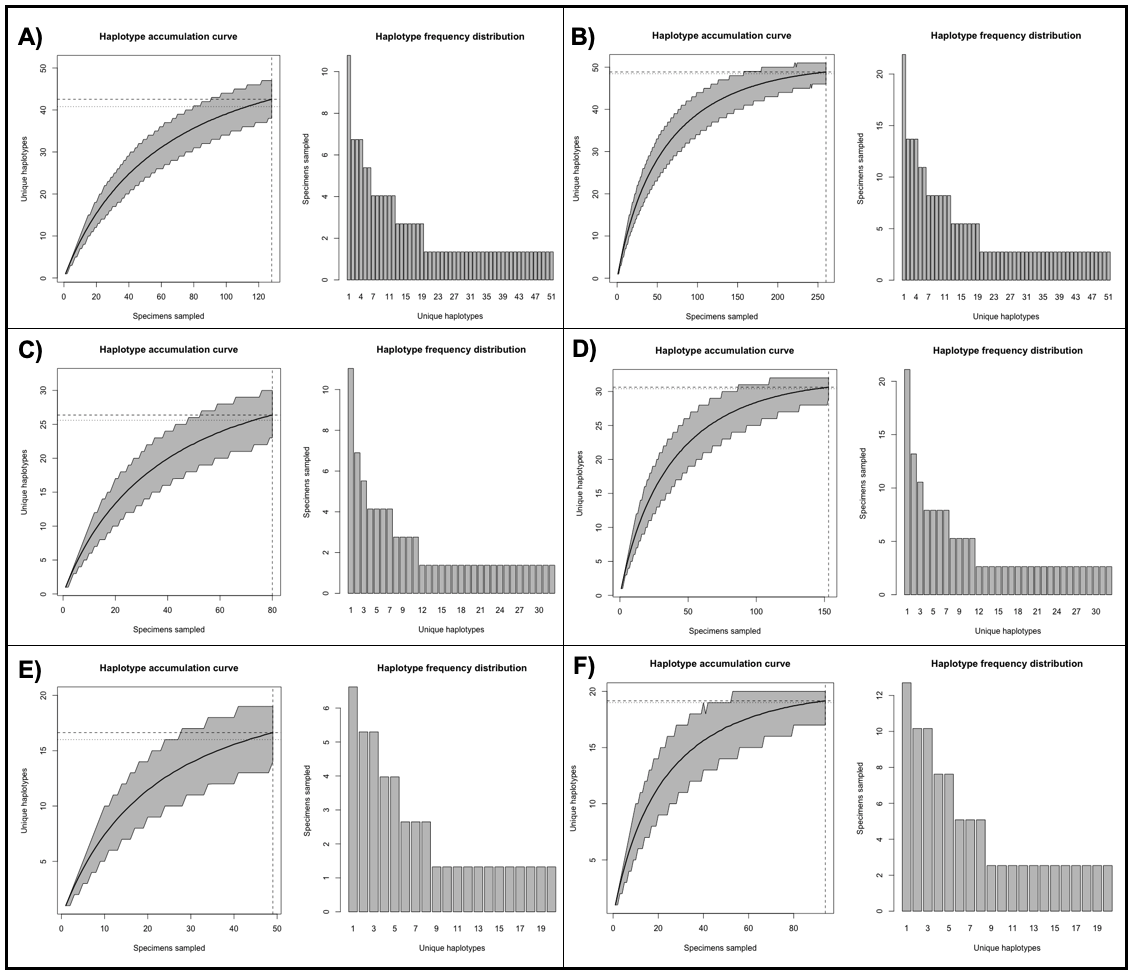


**Figure S4.** HACSim haplotype accumulation curves and hapltoye frequency distributions based on 778 bp cytochrome-b haplotypes for all sunda pangolin (*n*=95) at 80% (a) and 95% (b) haplotype recovery, sample from the ‘mainland, Sumatra and west/south Borneo’ clade’ (*n*=58) at 80% (c) and 95% (d) haplotype recovery, and samples from the ‘north Borneo and Java’ clade (*n*=37) at 80% (e) and 95% (f) haplotype recovery.

**
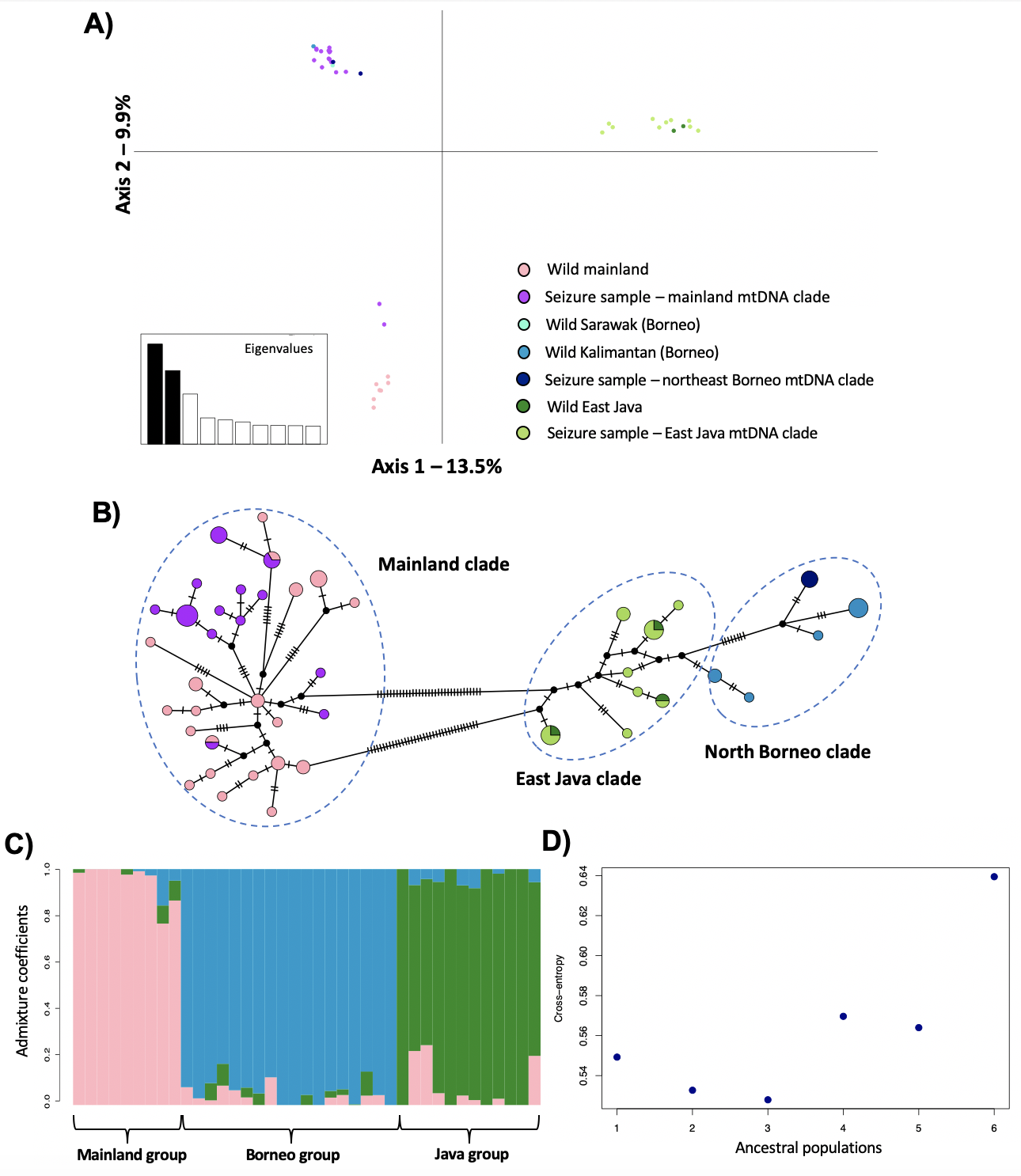
**

**Figure S5.** A) PCoA plot based on 39 Sunda pangolins and 8,911 SNPs from Nash et al. (2018). The dataset included wild sourced individuals and seized individuals that had a corresponding mtDNA haplotype that clearly clustered in one of the three mtDNA clades (indicated in the legend). B) TCS-based haplotype network for 74 Sunda pangolin samples based on 1519 bp of concatenated CO1 and cytochrome-b. Dashes on haplotype network branches represent substitutions, and the sizes of circles are proportional to the number of samples. The network includes all of the wild samples from Table S1, and the seizure samples included are the same individuals analysed in the PCoA (A). C) The sNMF analysis for *K*=3, based on the same 39 Sunda pangolin samples as the PCoA (A), and 4,929 SNPs (filtered for LD and HWE). D) Cross-entropy values for the sNMF analysis; lower values are considere more ‘optimal.’


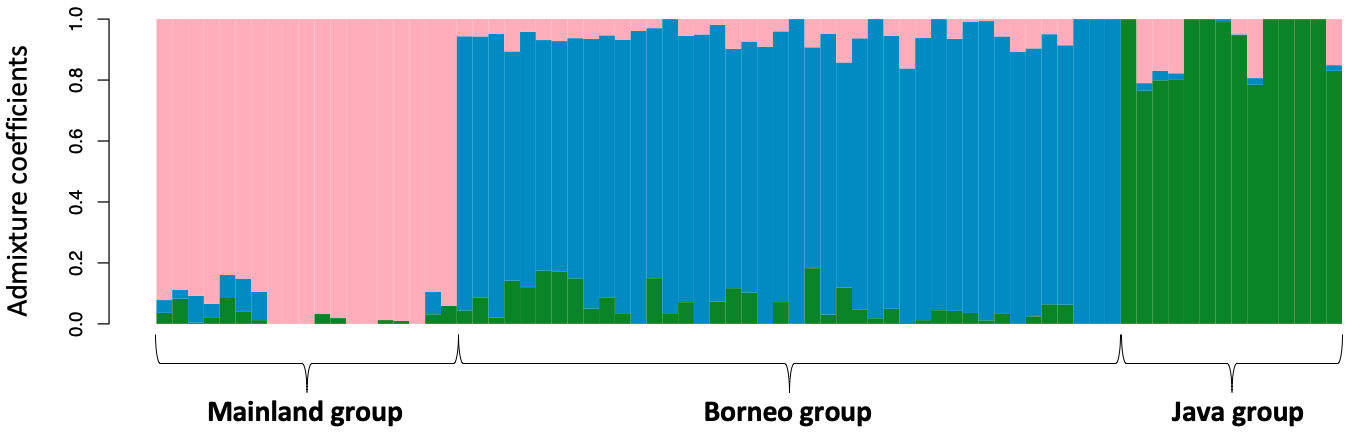


**Figure S6.** A sNMF analysis for *K*=3, based on 5,129 SNPs (filtered for LD and HWE) and 75 wild sourced Sunda pangolins and all seized individuals that clustered into the three distinct populations characterized by Nash et al. (2018) (i.e. the same samples as the PCoA in Figure 2c). The population delineations (i.e. mainland, Borneo and Java) inferred in this analysis and in Figure 2c (i.e. a more expansive dataset compared to Figure S5) were utilized in the subsequent genetic diversity and population diversity analyses (i.e. Table S5 and S6).

**Table S5.** Observed heterozygosity, expected heterozygosity, rarefied allelic richness and number of private alleles for the three putative Sunda pangolin populations based on 75 individuals and 5,129 SNPs. Seized individuals were assigned to populations based on clustering analyses (see Figure S6). Note, the private allele counts were not rarefied.

| **Putative population** | **SNPs** | **# of individuals** | **Observed heterozygosity** | **Expected heterozygosity** | **Allelic richness** | **Private allele count** |
| --- | --- | --- | --- | --- | --- | --- |
| Mainland | 5,129 | 19 | 0.123 | 0.127 | 7453.41 | 176 |
| Borneo |  | 42 | 0.177 | 0.175 | 8657.36 | 1707 |
| Java |  | 14 | 0.094 | 0.101 | 6875.66 | 165 |

**Table S6.** *F*_ST_ estimates between the three putative Sunda pangolin populations based on 75 individuals and 5,135 SNPs. Seized individuals were assigned to populations based on clustering analyses. All *F*_ST_ values were significant, as their associated confidence intervals did not encompass zero.

| **Mainland** | - |  |  |
| --- | --- | --- | --- |
| **Borneo** | 0.066 (0.062 – 0.070) | - |  |
| **Java** | 0.125 (0.117 – 0.133) | 0.093 (0.088 – 0.097) | - |
|  | **Mainland** | **Borneo** | **Java** |

**Supplementary Material References**

Gao, W. H., Lin, X. D., Chen, Y. M., Xie, C. G., Tan, Z. Z., Zhou, J. J., ... & Zhang, Y. Z. (2020). Newly identified viral genomes in pangolins with fatal disease. *Virus Evolution*, *6*, veaa020.

Gaubert, P., Antunes, A., Meng, H., Miao, L., Peigné, S., Justy, F., ... & Luo, S. J. (2018). The complete phylogeny of pangolins: scaling up resources for the molecular tracing of the most trafficked mammals on earth. *Journal of Heredity*, *109*, 347-359.

Hassanin, A., Hugot, J. P., & van Vuuren, B. J. (2015). Comparison of mitochondrial genome sequences of pangolins (Mammalia, Pholidota). *Comptes Rendus Biologies*, *338*, 260-265.

Hu, J. Y., Hao, Z. Q., Frantz, L., Wu, S. F., Chen, W., Jiang, Y. F., ... & Yu, L. (2020). Genomic consequences of population decline in critically endangered pangolins and their demographic histories. *National Science Review*, *7*, 798-814.

Hua, Y., Wang, J., An, F., Xu, J., Zhang, H., & Gu, H. (2020). Phylogenetic relationship of Chinese pangolin (*Manis pentadactyla aurita*) revealed by complete mitochondrial genome. *Mitochondrial DNA Part B*, *5*, 2523-2524.

Mason, V. C., Helgen, K. M., & Murphy, W. J. (2019). Comparative phylogeography of forest-dependent mammals reveals Paleo-forest corridors throughout Sundaland. *Journal of Heredity*, *110*, 158-172.

Nash, H. C., Low, G. W., Choo, S. W., Chong, J. L., Semiadi, G., Hari, R., ... & Rheindt, F. E. (2018). Conservation genomics reveals possible illegal trade routes and admixture across pangolin lineages in Southeast Asia. *Conservation Genetics*, *19*, 1083-1095.

Peng, M. S., Li, J. B., Cai, Z. F., Liu, H., Tang, X., Ying, R., ... & Zhang, Y. P. (2021). The high diversity of SARS-CoV-2-related coronaviruses in pangolins alerts potential ecological risks. *Zoological Research*, *42*, 834-844.

Wirdateti, Nugraha, R. T. P., Yulianto, & Semiadi, G. (2022). Identification of confiscated pangolin for conservation purposes through molecular approach. *Journal of Threatened Taxa*, *14*, 21127-21139.
